# Evaluation of Hematological Profiles and Monocyte Subpopulations in Water Buffalo Calves after Immunization with Two Different IBR Marker Vaccines and Subsequent Infection with *Bubaline alphaherpesvirus*-1

**DOI:** 10.3390/vaccines11091405

**Published:** 2023-08-23

**Authors:** Francesco Grandoni, Jamal Hussen, Federica Signorelli, Francesco Napolitano, Maria Carmela Scatà, Immacolata De Donato, Giovanna Cappelli, Giorgio Galiero, Carlo Grassi, Esterina De Carlo, Stefano Petrini, Giovanna De Matteis, Alessandra Martucciello

**Affiliations:** 1 Research Centre for Animal Production and Aquaculture, Consiglio per la Ricerca in Agricoltura e l’Analisi dell’Economia Agraria (CREA), 00015 Monterotondo, Italy; 2 Department of Microbiology, College of Veterinary Medicine, King Faisal University, Al-Ahsa 36362, Saudi Arabia; 3 National Reference Centre for Hygiene and Technologies of Water Buffalo Farming and Productions, Istituto Zooprofilattico Sperimentale del Mezzogiorno, 84132 Salerno, Italy; 4 National Reference Centre for Infectious Bovine Rhinotracheitis (IBR), Istituto Zooprofilattico Sperimentale Umbria-Marche, “Togo Rosati”, 06126 Perugia, Italy

**Keywords:** water buffalo, *Bubaline alphaherpesvirus-*1 (BuAHV-1), *Bovine alphaherpesvirus*-1 (BoAHV-1), IBR marker vaccines, monocyte subsets, flow cytometry

## Abstract

*Bubaline alphaherpesvirus-*1 (BuAHV-1) and *Bovine alphaherpesvirus-*1 (BoAHV-1) are respiratory viruses that can cause an infection known as “Infectious Bovine Rhinotracheitis” (IBR) in both water buffalo and bovine species. As the main disease control strategy, vaccination can protect animals from clinical disease through the development of specific humoral and cell-mediated immune responses. In the present study, the time-related circulatory kinetics of hematological profile and bubaline monocyte subsets have been investigated in vaccinated buffalo calves after challenge infections with BuAHV-1. Thirteen buffalo calves were selected and grouped into the VAX-1 group, which received an IBR-live-attenuated gE-/tk-deleted marker vaccine; the VAX-2 group, which received an IBR-inactivated gE-deleted marker vaccine; the CNT group, which remained an unvaccinated control. Fifty-five days after the first vaccination, the animals were infected with 5 × 10^5.00^ TCID_50_/mL of wild-type BuAHV-1 strain via the intranasal route. Whole blood samples were collected at 0, 2, 4, 7, 10, 15, 30, and 63 days post-challenge (PCDs) for the analysis of hematological profiles and the enumeration of monocyte subsets via flow cytometry. The analysis of leukocyte compositions revealed that neutrophils were the main leukocyte population, with a relative increase during the acute infection. On the other hand, a general decrease in the proportion of lymphocytes was observed early in the post-infection, both for the VAX-1 and VAX-2 groups, while in the CNT group, the decrease was observed later at +30 and +63 PCDs. An overall infection-induced increase in blood total monocytes was observed in all groups. The rise was especially marked in the animals vaccinated with an IBR-live-attenuated gE-/tK-deleted marker vaccine (VAX-1 group). A multicolor flow cytometry panel was used to identify the bubaline monocyte subpopulations (classical = cM; intermediate = intM; and non-classical = ncM) and to investigate their variations during BuAHV-1 infection. Our results showed an early increase in cMs followed by a second wave of intMs. This increase was observed mainly after stimulation with live-attenuated viruses in the VAX-1 group compared with the animals vaccinated with the inactivated vaccine or the non-vaccinated animal group. In summary, the present study characterized, for the first time, the hematological profile and distribution of blood monocyte subsets in vaccinated and non-vaccinated water buffalo in response to experimental infection with BuAHV-1. Although not experimentally proven, our results support the hypothesis of a linear developmental relationship between monocyte subsets.

## 1. Introduction

Water buffalo (*Bubalus bubalis*) is an important species because of its economic role in milk and meat production. As reported by the Food and Agriculture Organization [1], there are more than 200 million water buffalo in the world, with a continuous annual increase indicating growing economic interest in this species [2]. In Italy, the water buffalo population is estimated to be 350,000 animals, mainly reared in the central and southern parts of the country [3]. As two genera of the Bovidae family, water buffalo and bovines (*Bos taurus*) are closely related species, with similar disease resistance and susceptibility to a wide range of pathogens [2].

*Bubaline alphaherpesvirus-*1 (BuAHV-1) and *Bovine alphaherpesvirus-1* (BoAHV-1) are respiratory pathogens that can cause infection in both buffalo and bovine species [4].

In cattle, the infection often leads to a compromised host immune response that contributes to the development of a clinical disease known as “Infectious Bovine Rhinotracheitis” (IBR) [5]. Infection may take many forms in cattle, including respiratory infections, conjunctival infections, or infections of the male or female reproduction tract or the newborn animal. The respiratory form of BoAHV-1 is the most common and is characterized by clinical signs in the upper respiratory tract, such as (muco)purulent nasal discharge and dyspnea. In contrast, infection in water buffalo usually leads only to virological and serological positivity with asymptomatic infection [4]. However, infection in buffalo calves has been found to be associated with some respiratory and general illness symptoms like coughing, sneezing, wheezing, and nasal secretions, in addition to fever and loss of appetite. In addition, BoAHV-1 was recently isolated from an aborted buffalo fetus. Moreover, the reported susceptibility of buffalo to infection with BoAHV-1 supports their possible role as host/reservoirs of BoAHV-1 and creates a risk factor for cross-infections given the nature of buffalo and cattle farming, often being raised together in mixed farm operations [5,6].

BuAHV-1 appears to be widespread in buffalo farms in the Campania region [6]. The endemic presence of BuAHV-1 creates considerable economic problems and limits the trade of live animals. The main way to control the infection is vaccination, which protects animals from clinical disease and results in a good humoral and cell-mediated immune response.

In Europe, the control of IBR infections is associated with vaccination with inactivated and live-attenuated deleted (gene) products [7,8,9]. The most used marker products are based on the deletion of BoAHV-1 glycoprotein E (gE) and the thymidine kinase (tk) enzyme [10]. In the literature, there are few studies on the use of IBR commercial deleted marker vaccines for cattle used in buffalo species [3]. Furthermore, it is useful to evaluate the use of these recent double-deletion products to guarantee the application of vaccination protocols to buffalo species for which there are no specific products.

The immune response to BoAHV-1—as described in animals naturally or experimentally infected with the virulent BoAHV-1 virus or vaccinated with live or killed BoAHV-1 vaccines—is characterized by the development of both humoral and cell-mediated immune responses [11]. Previous research has been conducted to investigate the humoral immune response to BoAHV-1 infections via the detection of virus-specific antibodies in infected animal serum [12], while infection-induced alterations in immune cell compositions and functions are still not well investigated [13]. Cells of the mononuclear phagocytic system, including monocytes and their derived macrophages and dendritic cells, are key players in the innate immune response to BoAHV-1 infection and the subsequent initiation of the virus-specific adaptive immune response through antigen presentation to CD4-positive T helper cells and their subsequent polarization into different functional subsets (TH1, TH2, or TH17) [14,15]. For blood monocytes, which show high susceptibility to the virus, a special role in viral distribution to other tissues and disease pathogenesis has been reported [16]. In cows experimentally infected with BoAHV-1, a marked increase in the monocyte number, with an increased abundance of surface Fc receptors, was observed between days two to eight post-infection [17]. For several mammalian species, blood monocytes are not now considered a more homogenous population of circulation innate cells; they are rather classified into two or more subsets with specific phenotypes and functions. In bovine species, blood monocytes consist of three different subsets based on the expression of CD16 and CD14 [17]. Similarly, the heterogeneity of buffalo blood monocytes was recently reported with some similarities to and differences from bovine monocyte subsets. Using monoclonal antibodies to the signal regulatory protein (SIRP)-alpha protein (also known as CD172a), the CD14 LPS receptor, and the low-affinity CD16 FC receptor, bubaline classical (cM), intermediate (intM), and non-classical (ncM) monocyte subsets were characterized as CD14^+/++^/CD16^−/+^, CD14^+/low^/CD16^+/++^, and CD14^-/low^/CD16^++^, respectively [18].

Preliminary data on the use of BoAHV-1 acute infections have allowed us to highlight subsets of greater interest that differentiate vaccinated from unvaccinated buffalo [19]. In the present study, multicolor flow cytometry was used for the analysis of monocyte composition in the peripheral blood of water buffalo after experimental infection with BuAHV-1.

The aim of this study was to evaluate hematological profiles and dynamic variations in monocyte subpopulations in vaccinated and unvaccinated water buffaloes after acute infection with BuAHV-1.

## 2. Materials and Methods

### 2.1. Ethical Statement

The experimental protocol for the care, handling, and sampling of animals defined in the present study was approved by the Italian Ministry of Health (Authorization number n° 202/2021-PR). Furthermore, the authors complied with European legislation on the protection of animals used for scientific purposes, maintenance, and experimental protocols.

### 2.2. Virus

The wild-type (wt) BuAHV-1 strain was used for challenge infections [20,21]. The viruses were grown in Madin–Darby Bovine Kidney (MDBK) cells at 1.5 × 10^8.00^ median tissue culture infection doses (TCID_50_/mL), calculated using the Reed and Muench method [22]. All in vitro experiments using BuAHV-1 viruses were performed in a biosafety level-2 laboratory and facilities.

### 2.3. Experimental Design

Thirteen male buffalo calves, devoid of BoAHV-1 or BuAHV-1 neutralizing antibodies (NAs) evaluated according to Martucciello et al. [23], were enrolled in the study and divided into three groups kept in separate boxes.

Group VAX-1 (n = 4) received an IBR-live-attenuated gE-/tk-deleted marker vaccine administered via the intramuscular route.

**Figure 1:**
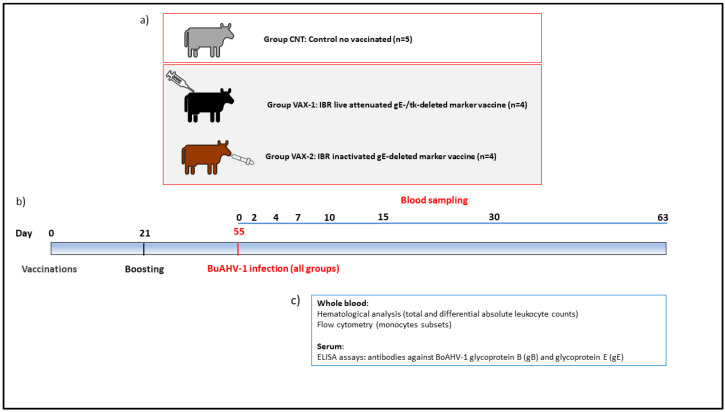
Experimental design. (**a**) Thirteen male buffalo calves were divided into three groups: VAX-1 (n = 4) received an IBR-live-attenuated gE-/tk-deleted marker vaccine administered via the intramuscular route; VAX-2 (n = 4) received an IBR-inactivated gE-deleted marker vaccine injected via the intranasal route; CNTs (n = 5) did not receive any vaccine and were used as the unvaccinated controls. (**b**) Two doses of these vaccines were administered to each animal 21 days apart. Fifty-five days after the first vaccination, all groups were subjected to challenge infections with the wt-BuAHV-1 strain. During the entire challenge period, whole blood and serum samples were collected from each water buffalo at 0, 2, 4, 7, 10, 15, 30, and 63 PCDs. (**c**) Whole blood was used to evaluate the hematological profile and monocyte subpopulations; serum samples were collected for ELISA assays.

Group VAX-2 (n = 4) received an IBR-inactivated gE-deleted marker vaccine injected via the intranasal route. The water buffalo calves (N = 5) that did not receive any vaccine were used as the unvaccinated controls (CNTs) (Figure 1a).

Both marker vaccines were commercially available at the time of the study and were administered via the route recommended by the manufacturers. Two doses (2 mL/each) of these vaccines were administered to each animal 21 days apart. After a 40-day acclimatization period, the buffalo received the first dose of the vaccine at the age of 4 months.

Fifty-five days after the first vaccination, all groups were subjected to challenge infections with the wt-BuAHV-1 strain. Each animal received 5 mL × 10^5.00^ TCID_50_/mL administered via the intranasal route (Figure 1b).

The animals were observed for 63 days post-challenge (PCDs), and rectal temperatures were recorded daily in the morning between 7:00 and 8:00 a.m. During this period, fever was confirmed when the rectal temperature was greater than 38.2 °C [24]. In addition, clinical evaluations were observed daily.

During the entire experimental period (0, 2, 4, 7, 10, 15, 30, 63 PCDs), whole blood and serum samples were collected from each water buffalo and used for hematological and serological (ELISA test) investigations, respectively (Figure 1c).

### 2.4. Hematological and Flow Cytometry Analysis

Whole blood samples were collected from the jugular vein in K_3_-EDTA and Li-Heparin test tubes (Vacuette^®^, Greiner Bio-One, Cassina de Pecchi, Italy) for hematological and flow cytometric analyses, respectively. Total and differential absolute leukocyte counts were performed using a hematology analyzer, Cell-Dyn 3700 SL (Abbott, Abbott Park, IL, USA), according to the standard operating procedure.

**Figure 2:**
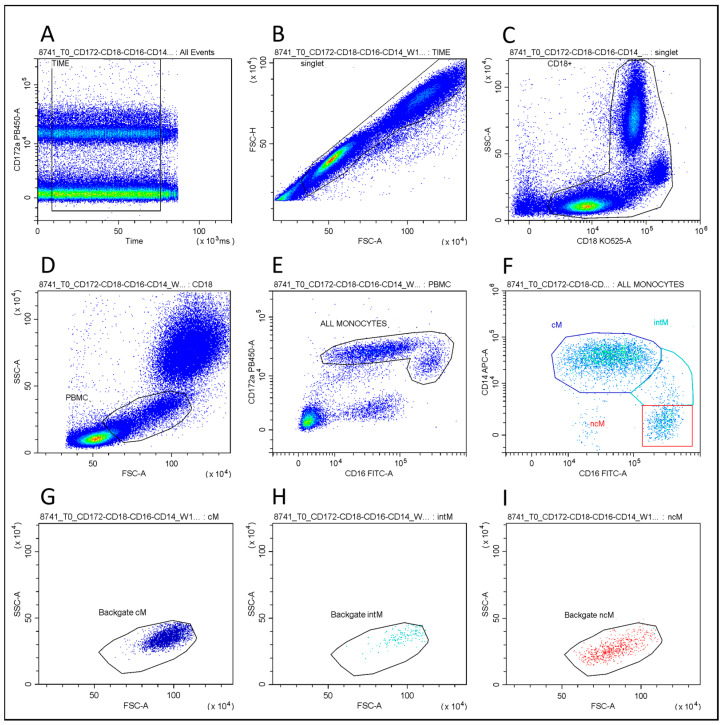
Flow-cytometric gating strategy used to identify monocyte subsets.. The gate “time” (in dot plot time vs. FL-PB450) was used to exclude event bursts (**A**). The gate “singlets” (FSC-A vs. FSC-H) was used to exclude doublets (**B**), while the gate “CD18^+^” (CD18 vs. SSC) was used to identify total leukocytes (**C**). The gate “PBMC” (**D**) was used to identify the respective subpopulation in the FSC vs. SSC dot plot, excluding debris, damaged and/or dead cells, and “small lymphocytes” (**D**). Applying this gate to the CD16 vs. CD172a dot plot (**E**), we identified the “all monocytes” gate, which was used to identify the three monocyte subsets in the CD16 vs. CD14 dot plot (**F**). Finally, backdating on the FSC vs. SSC dot plot (**G**–**I**) highlights the different physical characteristics of the three subsets and the overlap of ncMs with lymphocytes.

The cM, intM, and ncM subsets were detected using multicolor flow-cytometric panels: Pacific Blue anti-CD172 a (clone CC149; Biorad Laboratories, Hercules, CA, USA), BV510 anti-CD18 (clone 6.7; BD Pharmingen, Franklin Lakes, NJ, USA); FITC anti-CD16 (clone KD1; Bio-Rad Laboratories, CA, USA); and AF647 anti-CD14 (clone MM61 A, Washington State University, Pullman, WA, USA). Clones CC149 and MM61 a were conjugated in house as reported by Grandoni et al., 2023 [18]. All labeled samples were immediately acquired using a CytoFLEX flow cytometer, and the data were analyzed using the CytExpert software (Beckman Coulter, Brea, CA, USA). The absolute monocyte subset counts were performed using a dual-platform approach. In brief, they were calculated based on the flow-cytometric-assessed percent subpopulations within the leukocytes (CD18^+^ cells, Figure 2) and the absolute leukocyte count from a hematology cell analyzer.

### 2.5. ELISA Test

Serum samples collected from each animal were tested using two commercial ELISA tests (IDEXX IBR gB X3 Ab, Westbrook, ME, USA; IDEXX IBR gE Ab test, Westbrook, ME, USA) to detect antibodies to BoAHV-1 glycoprotein B (gB) and glycoprotein E (gE). Data were analyzed using Microplate Manager version 6 (Biorad Laboratories, Hercules, CA, USA). All results were interpreted according to the manufacturer’s instructions.

### 2.6. Statistical Analysis

All data were analyzed using the PROC MIXED procedure of SAS 9.4 (SAS Institute Inc., Cary, CA, USA) as follows: Y_ijkl_ = µ + G_i_ + D_j_ + GD_ij_ + b_jk_+ ε_ijkl_, where Y_ijkl_ is the dependent variable; µ is the overall mean; G_i_ is the fixed effect of the ith group (control, VAX-1, and VAX-2); D_j_ is the fixed effect of the jth day post-infection (0, 2, 4, 7, 10, 15, 30, and 63 PCDs); GD_ij_ is the fixed effect of the interaction between the ith group and jth day; b_jk_ is the random effect of the subject within the time j (k =1, …,13); and ε_ijkl_ is the random error. Differences were considered significant at *p* < 0.05.

## 3. Results

### 3.1. Clinical Observations

No clinical signs were observed after vaccination. In addition, after the challenge, buffaloes in the control group showed clinical respiratory signs such as a loss of nasal mucus, nasal mucosal lesions associated with mucopurulent exudate, and blood. In addition, rectal temperatures increased up to 39.1 °C from 4 to 11 PCDs. In contrast, no clinical signs and increased rectal temperatures were observed in groups VAX-1 and VAX-2.

### 3.2. Serological Investigations

Groups VAX-1 and VAX-2 were gB-positive on the day of the challenge (0 DPC). Conversely, the control group seroconverted to gB on PCD 10. However, group VAX-1 remained negative to gE during the entire experimental period, while group VAX-2 seroconverted to gE on PCD 30. On PCD 30, vaccinated animals were seropositive for gB-ELISA and negative for gE-ELISA (Table 1).

### 3.3. Relative and Absolute Count of the Main Populations of Blood Leukocytes

In Table 2, the measurements of each leukocyte population are compared between groups and during the whole period after the experimental infection of each group.

For each leukocyte population, values post-challenge (at 2, 4, 7, 10, 15, 30, and 63 PCDs) were compared with the pre-challenge values (at 0 PCD). In addition, for each leukocyte population and at each timepoint, values for the VAX-1 and VAX-2 groups were compared with the control group values.

The hematological analysis showed that the absolute count of leukocytes was significantly (*p* < 0.05) decreased in all three groups at PCD 15 with respect to day 0, while an increase was observed from PCD 30 to 63 PCD. The neutrophils were the main population that showed a relative increase during the time course of infection. In comparison with pre-challenge values on day 0, the increase in the fraction of neutrophils was significant at PCD 10 for the control and VAX-2 groups, at PCD 30 for all groups, and at PCD 63 only for the control group (*p* < 0.05). Furthermore, the absolute count of neutrophils showed a significant decrease at PCD 15 only in the VAX-2 group, followed by a significant increase at PCD 30 in all groups. Only for the control and VAX-2 groups the absolute neutrophil number remain significantly higher at PCD 63 with respect to day 0.

Compared with day 0, a general decrease in the lymphocyte percentage was observed early in the post-challenge time for the vaccinated groups: a significant (*p* < 0.05) decrease was observed in group VAX-2 at PCDs 2, 4, 7, 10, and 30; in group VAX-1 at PCDs 4, 7, and 30; and in the unvaccinated controls at PCD 30 and 63. The absolute count of lymphocytes showed an early decrease starting from PCD 2 to PCD 15, with significant differences at PCD 2 in the control and VAX-2 groups and in all groups at PCD 15 (*p* < 0.05).

The total monocytes showed a significant (*p* < 0.05) increase at PCD 4 (for both the absolute count and the relative percentage) and PCD 63 (only for the absolute count) only in group VAX-2 (Table 2).

The comparison of the three groups also revealed significant differences regarding the relative and absolute counts of the main leukocyte populations. Significant (*p* < 0.05) pre-infection differences between the groups were observed only in the baseline total count of neutrophils, with higher values in vaccinated groups compared with the control group. These differences maintained up to PCD 30.

An overall lower percentage of lymphocytes was observed in both vaccinated groups with respect to the control group, with significant differences between the VAX-1 and control groups only at PCDs 4 and 7.

**Figure 3:**
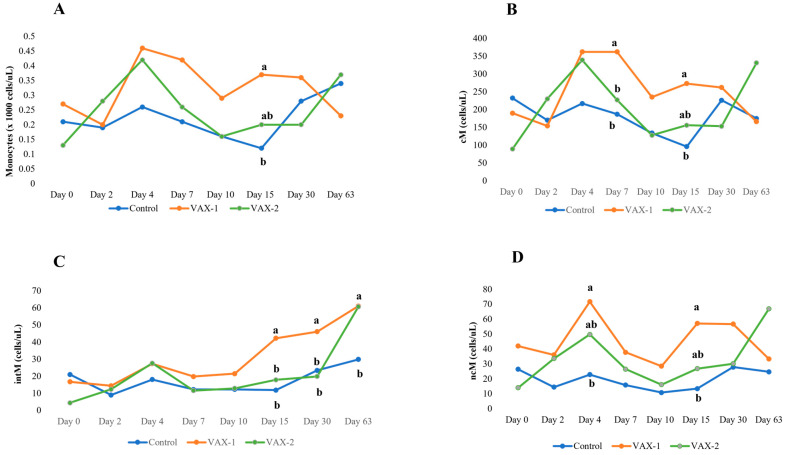
Time-related changes in the absolute count of total bubaline monocyte (**A**), classical monocyte (**B**), intermediate monocyte (**C**), and non-classical monocyte (**D**) subsets in vaccinated and unvaccinated buffalo calves after experimental infection with BuAHV-1. Significant differences among Groups at single time point (^a, b^); *p* < 0.05.

In addition, regarding the monocyte population, a significantly (*p* < 0.05) higher percentage and absolute count was observed at PCD 15 in the VAX-1 group compared with the control group (Figure 3).

### 3.4. Flow Cytometric Profiling of Bubaline Monocyte Subsets

A deep phenotyping of monocyte subpopulations was performed via flow cytometry (Figure 2). The percentage and absolute count of bubaline classical (cM), intermediate (intM), and non-classical (ncM) monocyte subsets were compared using the groups and during the whole experimental infection. Significant (*p* < 0.05) differences were found for each subset using the groups and during the post-challenge period (Table 3).

Compared with day 0, the percentage of cM cells increased significantly in the control group at PCDs 2 and 7, whereas the VAX-1 group showed a higher percentage at PCDs 7 and 10 and a lower percentage at PCD 63. The VAX-2 group showed significantly (*p* < 0.05) lower cM values at PCD 63.

On average, the BuAHV-1 infection resulted in an early increase in the absolute count of cMs at PCD 4 in both vaccinated groups and at PCD 7 only for the VAX-1 group, while a significant decrease was found at PCD 15 in the control group. Indeed, at these two timepoints, a significant difference was observed between the control and VAX-1 groups. No differences were observed between the two vaccinated groups (Table 3 and Figure 3).

Especially for the intM subset, noticeable differences were observed between the groups and the timepoints within each group. Compared with the pre-challenge values on day 0, a marked (*p* < 0.05) increase in the fraction of intMs was observed from PCD 15 to PCD 63 in the VAX-1 group and at PCDs 4 and 63 in the VAX-2 group with respect to day 0. Moreover, the VAX-1 group showed a higher absolute count of intMs compared with the VAX-2 and unvaccinated control groups during the whole post-challenge period. At day 63, both vaccinated groups showed significantly (*p* < 0.05) high absolute counts compared with the unvaccinated control group (Figure 2).

On average, the ncM subset highlighted an increase in the absolute counts in both vaccinated groups, although significant differences were found only in the VAX-2 group at PCDs 4 and 63 with respect to day 0. In the control group, a decrease in this subset was observed during the infection, but no significant differences were found at each timepoint.

An overall lower percentage of ncMs was observed in the control group during the whole experimental period compared with the two vaccinated groups. However, significant differences were only found between the control group and VAX-1 at PCDs 4 and 15 (Table 3 and Figure 3).

## 4. Discussion

*Bubaline alphaherpesvirus* 1, which is closely related to *Bovine alphaherpesvirus 1*, is responsible for clinical infections in water buffalo calves, representing a considerable economic threat to animal health and production [6,25]. Protective immunity to alphaherpesviruses depends on the activation of both humoral and cell-mediated immune responses.

In this study, two IBR marker vaccines were tested on the water buffalo species. The products induced neither adverse reactions nor clinical symptoms after their administration. In both groups, no clinical signs were observed during the entire experimental period. gB-ELISA antibodies were detected in the VAX-1 and VAX-2 groups on the day of the challenge, and the same antibodies were detected until the end of the experiments. Otherwise, the positivities against the gB protein were detected in the unvaccinated controls for the first time at PCD 10. These results are similar to those published by other authors [23].

Concerning the results obtained with the gE-ELISA test, the VAX-1 group was always negative throughout the experiment, whereas in the VAX-2 and control groups, seroconversion to this protein was observed on PCD 30. The serological results obtained in this study are similar to those published by other authors [19,23]. Martucciello et al. and Petrini et al. reported similar time courses for the humoral immunity of buffalo vaccinated and experimentally infected with BuAHV-1.

In the present study, the change in leukocyte compositions and the time-related circulatory kinetics of bubaline monocyte subsets were investigated after experimental challenges with BuAHV-1. Blood monocytes and their derived antigen-presenting cells, including macrophages and dendritic cells, are key players in innate immunity and the subsequent activation of virus-specific adaptive immune responses [26,27].

The analysis of leukocyte composition revealed significant infection-induced changes in all groups. The observed decrease in total leukocyte numbers in all groups at PCD 15 seems to be a result of the significant decrease in lymphocyte numbers at the same timepoint. This is in line with the reported leukopenia and lymphopenia associated with BoAHV-1 infection in bovine calves [28,29]. On the other hand, the rise in neutrophil numbers during the late post-infection time, which resulted in a leukocytosis between PCDs 30 and 63, seems to be in contrast to reported neutropenia after BoAHV-1 infection [30]. In that report, Molina et al. observed a decrease in the number of blood neutrophils in cows experimentally infected with BoAHV-1 starting on day 4 post-infection.

After their production in bone marrow, monocytes are released into the bloodstream, where they circulate for a few days before they migrate to the peripheral tissues. The relative composition of monocytes and the absolute count of their subsets in blood are mainly determined based on their production rate in the bone marrow and their migration rates into the peripheral tissues in response to infection or inflammation [31,32]. In the present study, although it was only tendential for all groups, the increase in the total monocyte number, which was observed at PCD 4, could be due to enhanced monocyte production in the bone marrow in response to infection stimuli. On the other hand, the following decrease starting at PCD 7 in the control group and the VAX-2 group and at PCD 10 in the VAX-1 group may indicate the enhanced migration of blood monocytes into the tissue.

In water buffalo, blood monocytes were recently classified into three different cell subsets based on the cell surface abundance of the CD14 LPS receptor and the CD16 FC receptor: CD14^+/++^CD16^−/+^ classical monocytes (cMs), CD14^+/low^CD16^+/++^ intermediate monocytes (intMs), and CD14^-/low^CD16^++^ non-classical (ncM) monocytes [18]. Although the dominant fraction of cMs (74–86% of total monocytes) with a minor population of intMs (5–8% of total monocytes) are in line with their distribution in bovine [33] and human blood [34], the frequency of bubaline ncMs (8–18% of total monocytes) seems to be higher than their bovine and human counterparts [33,34]. Further investigation is required to characterize the functional properties of bubaline monocyte subsets and to uncover possible correlations between species-specific monocyte compositions and the clinical relevance of monocyte subsets.

In the present study, the observed increase in the absolute number of intMs in the control and vaccinated groups indicates a special role for these cells in the immune response to infection with BuAHV-1. In humans and bovines, intM monocytes appear to be specialized in regulatory/anti-inflammatory functions and tissue repair, as well as antiviral responses and T cell immunomodulation. Their active involvement in several viral infections was recently reported [35,36,37].

Although the similar immunophenotypes of bovine and buffalo intMs may indicate similar functions, the functional properties of intMs in buffalo have not been investigated so far. Therefore, it can only be speculated that buffalo intMs are inflammatory monocytes with an important role in the immune response to viral infection. This is also supported by the observed higher increase in intMs after stimulation with live-attenuated viruses in the VAX-1 group compared with the animals vaccinated with inactivated vaccine or the non-vaccinated animal group. In addition, bovine and human intMs show enhanced expression levels of major histocompatibility complex (MHC)-class II molecules, giving them a crucial role in presenting antigens to and the polarization of helper T cells [33,38,39,40]. Therefore, the observed expansion in the number of buffalo intMs in the VAX-1 group may have contributed to the better immune response and antigen presentation to helper T cells in those animals.

Based on the gradual change in their phenotypes (expression of surface molecules) and functions (gene expression and antimicrobial effector functions), a developmental relationship was recently discussed for monocyte subsets [37]. Evidence exists from several in vivo and in vitro studies that bone marrow monocytes populate the bloodstream as cMs that differentiate into intMs and further into ncMs [41]. In the present study, the time course of monocyte composition—with an early increase in the number of cMs between PCDs 4 and 7, followed by their decrease starting on PCD 10, together with the observed late expansion in the intM subset starting on day 15—suggests a developmental relationship between the monocyte subsets in buffalo. Although further in vivo and in vitro studies are needed to confirm the developmental relationship between bubaline monocyte subsets, our results indicate that the monocyte response to BuAHV-1 is characterized by a first wave of cMs that populate the bloodstream followed by a second wave of intMs. However, further studies with extended sampling periods are required to investigate whether the observed decline in cMs is due to the development of these cells into intMs and whether those intMs will further differentiate into ncMs, resulting in a wave of ncMs during the late phase of infection.

## 5. Conclusions

In summary, the present study characterized, for the first time, the time course of the hematological profile and distribution of blood monocyte subsets in vaccinated and non-vaccinated water buffalo in response to experimental infection with BuAHV-1. In all groups, BuAHV-1 infection was associated with early leukopenia and lymphopenia at PCD 15 followed by neutrophilia and leukocytosis starting at PCD 30. An overall infection-induced increase in blood monocytes was also observed in all groups. The analysis of monocyte subsets revealed an early increase in cMs followed by a second wave of intMs. The increase was especially marked in the animals vaccinated with live-attenuated virus vaccines. Although not experimentally proven, our results support the hypothesis of a linear developmental relationship between monocyte subsets.

## Figures and Tables

**Table 1:** Antibody response of buffalo calves immunized with two different IBR-live-deleted marker vaccines and challenge infected with the wt-BuAHV-1 strain.

Group	Test	Post-Challenge Day (PCD)							
		0	2	4	7	10	15	30	63
VAX-1	gE-ELISA ^1^	−	−	−	−	−	−	−	−
	gB-ELISA ^2^	+	+	+	+	+	+	+	+
VAX-2	gE-ELISA ^1^	−	−	−	−	−	−	+	+
	gB-ELISA ^2^	+	+	+	+	+	+	+	+
CNT	gE-ELISA ^1^	−	−	−	−	−	−	+	+
	gB-ELISA ^2^	−	−	−	−	+	+	+	+

PCD 0 corresponds to 55 post vaccination days ^1^ IDEXX IBR gB X3 Ab, Westbrook, ME, USA; ^2^ IDEXX IBR gE Ab test, Westbrook, ME, USA.

**Table 2:** Comparison of total and differential leukocyte percentages and absolute counts (mean ± SEM) in three experimental groups.

Post-Challenge Day (PCD)									
	Group	0	2	4	7	10	15	30	63
Leukocytes (×1000 cells/μL)	CNT	**4.90 ± 0.61 ^B^**	4.28 ± 0.61	4.68 ± 0.61	4.59 ± 0.61	4.74 ± 0.61	**3.70 ± 0.61 ^C^**	**5.96 ± 0.61 ^A^**	**6.15 ± 0.61 ^A^**
	VAX-1	**5.51 ± 0.68 ^B^**	4.88 ± 0.68	5.44 ± 0.68	5.69 ± 0.68	5.21 ± 0.68	**4.48 ± 0.68 ^C^**	**7.16 ± 0.68 ^A^**	**6.22 ± 0.68**
	VAX-2	**5.04 ± 0.68 ^B^**	4.45 ± 0.68	4.66 ± 0.68	5.15 ± 0.68	5.15 ± 0.68	**3.73 ± 0.68 ^C^**	**6.90 ± 0.68 ^A^**	**6.69 ± 0.68 ^A^**
Neutrophils (%)	CNT	**41.0 ± 4.1 ^B^**	45.2 ± 4.1	43.5 ± 4.1	46.3 ± 4.1	**49.3 ± 4.1 ^A^**	43.9 ± 4.1	**52.7 ± 4.1 ^A^**	**50.3 ± 4.1 ^A^**
	VAX-1	**50.7 ± 4.5 ^B^**	53.5 ± 4.5	52.9 ± 4.5	56.6 ± 4.5	54.1 ± 4.5	51.0 ± 4.5	**57.7 ± 4.5 ^A^**	52.0 ± 4.5
	VAX-2	**52.1 ± 4.5 ^B^**	54.4 ± 4.5	49.6 ± 4.5	57.7 ± 4.5	**60.5 ± 4.5 ^A^**	49.8 ± 4.5	**61.2 ± 4.5 ^A^**	56.3 ± 4.5
Neutrophils (cells/μL)	CNT	**1.91 ± 0.27 ^b-B^**	1.86 ± 0.27	**1.95 ± 0.27 ^b^**	**2.02 ± 0.27 ^b^**	**2.24 ± 0.27 ^b^**	1.53 ± 0.27	**3.12 ± 0.27 ^b-A^**	**3.03 ± 0.27 ^A^**
	VAX-1	**2.78 ± 0.30 ^a-B^**	2.60 ± 0.30	**2.87 ± 0.30 ^a^**	**3.22 ± 0.30 ^a^**	**2.80 ± 0.30 ^ab^**	2.28 ± 0.30	**4.13 ± 0.30 ^a-A^**	3.23 ± 0.30
	VAX-2	**2.61 ± 0.30 ^ab-B^**	2.41 ± 0.30	**2.32 ± 0.30 ^ab^**	**2.93 ± 0.30 ^a^**	**3.10 ± 0.30 ^a^**	**1.85 ± 0.30 ^C^**	**4.19 ± 0.30 ^a-A^**	**3.75 ± 0.30 ^A^**
Lymphocytes (%)	CNT	**50.9 ± 4.2 ^A^**	49.5 ± 4.2	**50.3 ± 4.2 ^a^**	**46.0 ± 4.2 ^a^**	46.1 ± 4.2	51.3 ± 4.2	**41.3 ± 4.2 ^B^**	**42.5 ± 4.2 ^B^**
	VAX-1	**44.0 ± 4.7 ^A^**	41.5 ± 4.7	**36.9 ± 4.7 ^b-B^**	**34.6 ± 4.7 ^b-B^**	39.2 ± 4.7	39.6 ± 4.7	**35.9 ± 4.7 ^B^**	43.6 ± 4.7
	VAX-2	**45.2 ± 4.7 ^A^**	**38.6 ± 4.7 ^B^**	**40.6 ± 4.7 ^ab^**	**36.0 ± 4.7 ^ab-B^**	**35.9 ± 4.7 ^B^**	44.4 ± 4.7	**34.3 ± 4.7 ^B^**	37.5 ± 4.7
Lymphocytes (cells/μL)	CNT	**2.65 ± 0.41 ^A^**	**2.19 ± 0.41 ^B^**	2.44 ± 0.41	2.31 ± 0.41	2.29 ± 0.41	**2.01 ± 0.41 ^B^**	2.51 ± 0.41	2.70 ± 0.41
	VAX-1	**2.43 ± 0.46 ^A^**	2.02 ± 0.46	2.01 ± 0.46	1.98 ± 0.46	2.07 ± 0.46	**1.79 ± 0.46 ^B^**	2.58 ± 0.46	2.71 ± 0.46
	VAX-2	**2.30 ± 0.46 ^A^**	**1.72 ± 0.46 ^B^**	1.90 ± 0.46	1.92 ± 0.46	1.86 ± 0.46	**1.67 ± 0.46 ^B^**	2.40 ± 0.46	2.50 ± 0.46
Monocytes (%)	CNT	5.1 ± 1.4	4.2 ± 1.4	5.6 ± 1.4	5.6 ± 1.4	3.6 ± 1.4	**3.6 ± 1.4 ^b^**	5.2 ± 1.4	6.0 ± 1.4
	VAX-1	4.6 ± 1.6	4.0 ± 1.6	8.6 ± 1.6	7.3 ± 1.6	5.7 ± 1.6	**8.2 ± 1.6 ^a^**	5.2 ± 1.6	3.8 ± 1.6
	VAX-2	**2.7 ± 1.6 ^B^**	6.2 ± 1.6	**9.2 ± 1.6 ^A^**	5.6 ± 1.6	3.1 ± 1.6	**5.3 ± 1.6 ^ab^**	3.0 ± 1.6	5.3 ± 1.6
Monocytes (×1000 cells/μL)	CNT	0.21 ± 0.07	0.19 ± 0.07	0.26 ± 0.07	0.21 ± 0.07	0.16 ± 0.07	**0.12 ± 0.07 ^b^**	0.28 ± 0.07	0.34 ± 0.07
	VAX-1	0.27 ± 0.08	0.20 ± 0.06	0.46 ± 0.08	0.42 ± 0.08	0.29 ± 0.08	**0.37 ± 0.08 ^a^**	0.36 ± 0.08	0.23 ± 0.08
	VAX-2	**0.13 ± 0.08** ^B^	0.28 ± 0.08	**0.42 ± 0.08 ^A^**	0.26 ± 0.08	0.16 ± 0.08	**0.20 ± 0.08 ^ab^**	0.20 ± 0.08	**0.37 ± 0.08 ^A^**

Significant differences between groups at single timepoint (^a,b^); significant differences between PCD 0 and a single timepoint (^A,B,C^); *p* < 0.05.

**Table 3:** Comparison of absolute counts (mean ± SEM) of monocyte subsets evaluated via flow cytometry.

Post-Challenge Day (PCD)									
	Group	0	2	4	7	10	15	30	63
cM cells (%)	CNT	**79.9 ± 3.3 ^B^**	**86.8 ± 3.3 ^a-A^**	84.1 ± 3.3	**87.5 ± 3.3 ^A^**	84.4 ± 3.3	81.1 ± 3.3	81.1 ± 3.3	**81.8 ± 4.1 ^a^**
	VAX-1	**75.6 ± 3.6 ^B^**	**74.0 ± 3.6 ^b^**	78.9 ± 3.6	**86.2 ± 3.6 ^A^**	**83.7 ± 3.6 ^A^**	75.9 ± 3.6	74.4 ± 3.6	**65.8 ± 4.3 ^b-C^**
	VAX-2	**81.2 ± 3.6 ^A^**	**83.6 ± 3.6 ^ab^**	82.4 ± 3.6	84.8 ± 3.6	81.5 ± 3.6	75.7 ± 3.6	80.1 ± 3.6	**73.2 ± 3.9 ^ab-B^**
cM cells (cells/μL)	CNT	**232 ± 53 ^A^**	170 ± 53	217 ± 53	**187 ± 53 ^b^**	134 ± 53	**96 ± 53 ^b-B^**	226 ± 53	175 ± 83
	VAX-1	**190 ± 60 ^B^**	154 ± 60	**362 ± 60 ^A^**	**362 ± 60 ^a-A^**	235 ± 60	**273 ± 60 ^a^**	262 ± 60	166 ± 83
	VAX-2	**89 ± 60 ^B^**	230 ± 60	**339 ± 60 ^A^**	**227 ± 60 ^ab^**	128 ± 60	**156 ± 60 ^ab^**	153 ± 60	**331 ± 68 ^A^**
intM cells (%)	CNT	7.3 ± 1.8	5.1 ± 1.8	6.9 ± 1.8	5.8 ± 1.8	8.6 ± 1.8	9.1 ± 1.8	8.5 ± 1.8	**10.3 ± 2.4 ^b^**
	VAX-1	**6.8 ± 2.0 ^B^**	8.2 ± 2.0	6.0 ± 2.0	4.7 ± 2.0	6.4 ± 2.0	**11.3 ± 2.0 ^A^**	**12.1 ± 2.0 ^A^**	**20.6 ± 2.5 ^a-A^**
	VAX-2	**5.0 ± 2.0 ^B^**	4.7 ± 2.0	6.6 ± 2.0	5.0 ± 2.0	8.4 ± 2.0	**9.0 ± 2.0 ^A^**	8.3 ± 2.0	**12.8 ± 2.2 ^b-A^**
intM cells (cells/μL)	CNT	20.96 ± 7.97	8.96 ± 7.97	18.05 ± 7.97	12.19 ± 7.97	12.28 ± 7.97	**11.83 ± 7.97 ^b^**	**23.38 ± 7.97 ^b^**	**29.85 ± 11.43 ^b^**
	VAX-1	**16.71 ± 8.91 ^B^**	14.37 ± 8.91	27.28 ± 8.91	19.78 ± 8.91	21.47 ± 8.91	**42.23 ± 8.91 ^a-A^**	**46.04 ± 8.91 ^a-A^**	**61.07 ± 11.63 ^a-A^**
	VAX-2	**4.36 ± 8.91 ^B^**	12.29 ± 8.91	**27.58 ± 8.91 ^A^**	11.56 ± 8.91	12.86 ± 8.91	**17.86 ± 8.91 ^b^**	**19.83 ± 8.91 ^b^**	**60.69 ± 9.90 ^a-A^**
ncM cells (%)	CNT	**13.0 ± 2.4 ^A^**	**8.2 ± 2.4 ^b^**	9.1 ± 2.4	**6.8 ± 2.4 ^B^**	**7.0 ± 2.4 ^B^**	10.2 ± 2.4	10.5 ± 2.4	8.3 ± 3.2
	VAX-1	**17.8 ± 2.6 ^A^**	**18.0 ± 2.6 ^a^**	15.2 ± 2.6	**9.1 ± 2.6 ^B^**	**9.9 ± 2.6 ^B^**	13.3 ± 2.6	13.6 ± 2.6	**12.2 ± 3.3 ^B^**
	VAX-2	14.0 ± 2.6	**11.9 ± 2.6 ^ab^**	11.1 ± 2.6	10.2 ± 2.6	10.1 ± 2.6	15.4 ± 2.6	11.8 ± 2.6	13.9 ± 2.9
ncM cells (cells/μL)	CNT	26.39 ± 11.75	14.45 ± 11.75	**22.79 ± 11.75 ^b^**	15.80 ± 11.75	10.81 ± 11.75	**13.41 ± 11.75 ^b^**	27.85 ± 11.75	24.65 ± 17.20
	VAX-1	41.96 ± 13.13	36.06 ± 13.13	**71.65 ± 13.13 ^a^**	37.74 ± 13.13	28.50 ± 13.13	**57.08 ± 13.13 ^a^**	56.66 ± 13.13	33.27 ± 17.44
	VAX-2	**14.04 ± 13.13 ^B^**	33.58 ± 13.13	**49.71 ± 13.13 ^ab-A^**	26.48 ± 13.13	16.06 ± 13.13	**26.79 ± 13.13 ^ab^**	30.12 ± 13.13	**66.94 ± 14.71 ^A^**

Significant differences between groups at a single timepoint (^a,b^); significant differences between PCD 0 and a single timepoint (^A,B,C^); *p* < 0.05.

## Data Availability

The original contributions presented in the study are included in the article. Further inquiries can be directed to the corresponding authors.
